# Making collaboration explicit through collaborative, diagnostic, and intervention activities: foundations for interprofessional education

**DOI:** 10.3389/fmed.2026.1907531

**Published:** 2026-10-23

**Authors:** Matthias J. Witti, Anna Weiß, Melanie Bauer, Eva Baumann, Michaela Schunk, Matthias Stadler, Constanze Richters

**Affiliations:** 1 Institute of Medical Education, LMU University Hospital, LMU Medizin, Ludwig-Maximilians-Universität München, Munich, Germany; 2 Catholic University of Applied Sciences Munich, Munich, Germany; 3 Rosenheim University of Applied Sciences, Rosenheim, Germany

**Keywords:** diagnostic reasoning, interprofessional collaborative practice, interprofessional education, intervention reasoning, wound care

## Abstract

**Introduction:**

Interprofessional collaborative practice (ICP) is essential for high-quality healthcare, especially in complex care situations. To support learners in responding to the demands of ICP, interprofessional education (IPE) must help them recognize critical points for collaboration and engage in diagnostic and intervention reasoning across professional boundaries. This study uses the context of wound care to illustrate where and how collaboration becomes critical, and to examine how related reasoning activities unfold.

**Method:**

A qualitative expert elicitation with supplementary audio-recorded reflections was conducted. Six experts provided 18 case-based written and verbal reflections on three simulated patient scenarios. The material was analyzed using content-structuring qualitative content analysis.

**Results:**

Eight critical collaboration points were identified, including pre-briefing, wound assessment, and follow-up care. Across all points, professionals described distinct diagnostic activities (DAs), such as gathering and evaluating evidence; intervention activities (IAs), such as planning and adjusting treatment; and collaborative activities (CAs), such as information sharing, negotiation, and coordination. These activity types occurred in dynamic, iterative patterns and were often situated at moments that experts conceptually described as requiring interprofessional coordination—a perspective aligned with the notion of “noticing.” Structuring these collaboration points into CAs, DAs, and IAs provides a functional lens to describe and support collaborative reasoning in practice.

**Conclusions:**

The findings offer a foundation for designing simulation-based IPE, structuring feedback, and specifying interprofessional contributions in assessment frameworks such as Entrustable Professional Activities (EPAs). Further research can explore the transferability of this approach to other clinical contexts and examine its integration into teaching, supervision, and assessment.

## 1 Introduction

Interprofessional collaborative practice (ICP) plays a key role in ensuring high-quality healthcare (1, 2), particularly in complex care situations such as wound care (3). In ICP, healthcare professionals with different perspectives work together to solve shared problems and make joint decisions to optimize patient care (4). To prepare students for these demands, medical, nursing, and clinical pharmacy education increasingly emphasize early ICP training (5–7).

A key component of ICP is collaborative diagnostic and management reasoning (8). Management reasoning encompasses decisions about treatment, follow-up, and resource allocation, including intervention reasoning. These processes involve diagnostic activities (DAs), such as generating and evaluating evidence and hypotheses (9), and intervention activities (IAs), such as generating, selecting, implementing, evaluating and adjusting treatment measures (8). These activities are shaped by collaborative activities (CAs), including joint information processing, negotiating treatment plans, coordinating responsibilities, and regulating team actions (10, 11). In clinical settings, CAs, DAs, and IAs unfold in iterative, interdependent cycles rather than in isolation, as decisions and actions are continuously re-evaluated and adapted (8). To better understand these dynamics, prior work has begun to capture them in a process-oriented model of collaborative reasoning, which conceptualizes DAs, IAs, and CAs as interdependent and temporally evolving components of team-based problem solving. However, this line of research has primarily focused on activity patterns and transitions in simulation-based settings, leaving open how these processes are structured and experienced in real clinical practice (12).

Collaboration is often triggered when professionals recognize that a situation requires input across disciplinary boundaries. This shared recognition can be described as *noticing*—the joint identification of moments (e.g., changes in a wound's condition) that call for interprofessional coordination and action (10, 11).

Interprofessional education (IPE) must therefore strengthen learners' ability to identify critical points for collaboration and engage in associated diagnostic and intervention reasoning (7, 11). However, empirical insight is lacking into where exactly collaboration becomes critical in clinical practice, how professionals perceive these moments, and how they can be described in a way that supports systematic teaching and assessment in IPE.

Wound care provides a relevant setting, requiring shared decisions and coordinated implementation across professions such as nursing, medicine and pharmacy (13). In these situations, collaborative reasoning and shared responsibility are not only desirable, but essential for effective patient care (14).

Despite its importance, little is known about how interprofessional collaboration shapes diagnostic and intervention processes in wound care, and how these processes can be systematically supported in IPE (5, 6, 15). While research to date has focused primarily on diagnostic reasoning (9, 16, 17), which has also been investigated in the collaborative context (18, 19), decision-making beyond diagnosis, particularly management-related aspects such as treatment planning, prioritization, and coordination, has received comparatively little explicit attention in medical education research (19, 20). Within this broader perspective, intervention reasoning can be understood as a core component of management reasoning, focusing on the selection, implementation, and adaptation of treatment (8). However, intervention reasoning differs from diagnostic reasoning in that it poses different cognitive and social demands and is situated (21).

This is particularly evident in wound care, where diagnostic assessments and therapeutic decisions are tightly interwoven and distributed across professions. Iterative adjustments are often required, and the effectiveness of care depends on how well CAs, DAs, and IAs are aligned (8).

At the same time, wound care remains underrepresented in structured IPE formats (3, 22–24) and validated tools for assessing individual and collaborative performance are lacking. Entrustable Professional Activities (EPAs) offer a promising framework for competency-based assessment (25–28). EPAs define complex professional tasks that learners can be entrusted with as they gain experience and competence. Assessment within this framework is grounded in observable behavior, including how learners contribute to collaborative action within the team (25). However, current EPA descriptions often remain vague when it comes to specifying the roles and contributions of different professions within shared clinical tasks, particularly regarding how ICP is enacted (29).

A structured classification into DAs, IAs, and CAs could help address this gap. By differentiating the types of professional contributions that occur within a shared task, this structure may support clearer alignment of EPA definitions with profession-specific roles, provide a basis for more targeted feedback, and improve the precision of IPE-related assessment formats.

To explore this gap, we investigated how experienced professionals from medicine, nursing, and clinical pharmacy describe collaboration in wound care. By analyzing their perspectives, we aimed to identify where collaboration becomes critical, what types of reasoning and interaction occur in these situations, and how these can be structured into DAs, IAs, and CAs. This structuring provides a functional lens on collaboration that can inform IPE formats, simulation designs, and assessment tools.

This study uses wound care as a prototypical example of complex interprofessional collaboration to investigate how professionals describe critical moments of collaboration and what kinds of reasoning activities unfold in these situations. Specifically, we aim to identify the points in the care process where professionals recognize the need for collaboration (*noticing*) and to analyze what kinds of activities (CA, DAs, IAs) unfold at those points. By structuring these collaboration episodes along the functional dimensions of CAs, DAs, and IAs, the study seeks to contribute to a clearer understanding of how interprofessional reasoning unfolds in practice. It provides a basis not only for refining IPE formats in wound care, but also for advancing the development of teaching, feedback, and assessment practices in interprofessional medical education more broadly.

The following research questions (RQs) were investigated:

**RQ1:** What are the critical points in collaboration (noticing) during interprofessional wound care as an example of complex IPE?
**RQ2:** Which CAs (e.g., coordination, negotiation, joint information processing) occur at these points?
**RQ3:** How are these activities related to DAs and IAs?

## 2 Materials and methods

### 2.1 Study design

A qualitative expert elicitation (30) with supplementary audio-recorded reflections was conducted from July to October 2024 to explore when and what information the professions of medicine, nursing, and pharmacy should exchange during wound care in Germany. The study is reported in accordance with COREQ (31).

Ethical approval was obtained from the responsible review board of the Faculty of Medicine, LMU Munich.

### 2.2 Setting and study context

The study formed part of a funded teaching project comprising two phases. Before expert verification, the learning objectives were identified based on the national curricula of the three participating professions. Based on these learning objectives, three paper-based clinical patient cases were developed, representing different patient histories and wound types: pressure ulcers, leg ulcers, and diabetic foot syndrome. For each case, relevant work steps and treatment options for interprofessional wound care were derived and systematically compiled from national and international guidelines and arranged in the sequence of the wound care process, resulting in about 100 successive work steps per case. Each case included a photograph of the wound.

In phase one, experts reviewed the paper-based clinical patient cases, the derived work steps, and the treatment options. In phase two, experts reviewed critical points of collaboration between the participating professions that, from their perspective, are necessary to foster collaborative diagnostic and intervention skills.

### 2.3 Data collection

Data were collected using a structured expert elicitation combining written responses and audio-recorded reflections. The guide for the expert elicitation was created using the method according to Helfferich (32). The guide with instructions comprised three working documents with work instructions on learning objectives, questions on practical relevance and authenticity for three paper-based clinical patient cases as well as instructions for confirming or correcting work steps and for stating when and what information should be exchanged between the professions. For every work step, experts could enter treatment recommendations, common treatment errors and moments of interprofessional exchange, the latter defined as the point of consultation of the other professions, the exchange of information, shared goals, joint performance and role clarification. The learning objectives were assessed across all cases. In addition to the written assessment, experts were asked to record short audio reflections at eight predefined places per case along this sequence of work steps, explaining and justifying their decisions (33). The guide was piloted in advance with a physician, a nurse and a clinical pharmacist.

Data collection took place remotely, with experts completing all materials independently in professional or domestic settings and without researchers present. Questions could be sent to AW via email or telephone. All experts received the identical materials in the same order, were briefed orally on the procedure beforehand, and received a separate written instruction for recording the think-aloud reflections. Within these places, the wording and internal structure of each spoken reflection were left to the experts. Experts were instructed to respond from their own professional perspective and were not asked to simulate the perspectives of the other professions. Due to strong reactivity, this approach supported systematic reflection on complex practice-oriented topics such as interprofessional wound care (34, 35). Each patient case was allocated 60 min. Each participant required approximately 180 min. Audio-recorded reflections were recorded digitally and sent encrypted to AW. Each expert completed all three cases, resulting in 18 case analyses.

### 2.4 Sample and participant recruitment

We sought experts who could provide competent information to close existing knowledge gaps and to evaluate and communicate the complex care process for interprofessional wound care in a practical manner. The sample was selected on the basis of predefined criteria. We included physicians, nurses and clinical pharmacists with at least ten years of professional experience who are certified wound experts, have teaching experience, have scientific publications in the field of wound research and have specific prior knowledge in the areas of ICP and IPE. An initial selection of the sample was based on literature. Relevant specialist publications were consulted. This method was combined with snowball sampling to use existing and new relationships in the setting (36).

AW conducted data collection and contacted all potential experts personally by e-mail and sent them a personal letter with a brief description of our research project in advance. Experts were informed about the researchers' professional backgrounds, the aims of the study and the researchers' involvement in the broader teaching project prior to participation. Nine experts were invited to participate in the study. Six of them agreed and completed it, while three did not respond or declined. There were no prior hierarchical relationships between the researchers and experts, and interactions were limited to study-related communication.

With six experts, the final sample size was considered appropriate for an expert elicitation approach, which prioritizes depth and domain-specific expertise over representativeness. Experts were treated as contributors to interpretive reflection, not solely as sources of domain knowledge (37). Each participant analyzed three patient cases, yielding a total of 18 qualitative, case-based datasets that formed the basis for a systematic, cross-case comparison. Following iterative comparative analysis across cases, data sufficiency was assumed when no substantially new aspects of collaborative processes emerged.

### 2.5 Analysis

For data processing, AW transcribed the audio-recorded reflections verbatim (33). All written and audio-recorded reflection data were anonymized in accordance with the criteria of Saunders et al. (38) All material was analyzed using a content-structuring qualitative content analysis according to Kuckartz (39) conducted by AW and MJW. The data analysis software MAXQDA Analytics Pro 2024 (version 24.8.0) was used. The categories were formed both *a priori* on the basis of the guide for the expert elicitation and inductively from the material. After coding the complete material with the differentiated category system, AW and MJW analyzed the main categories and subcategories using summary grids and summary tables (39) (see Supplementary Appendix A Analysis Example). The results of the analysis were then assigned to the CAs, DAs and IAs at segment level (sense units). We used the definitions provided by Meier et al. (10) and Witti et al. (11) to assign CAs. We assigned DAs based on Fischer et al. (9) and IAs based on Richters et al. (8). Supplementary Appendix C outlines the analytic framework used to categorize DAs and IAs in our summarized expert material. While all activity assignments in this study were made at the level of synthesized category summaries, we also provide indicative behavioral markers that may guide future research aiming to code these activities directly in transcripts or simulations (see Supplementary Appendix C Analytic Framework).

### 2.6 Reflexivity

Our research team comprised members with diverse professional, disciplinary and methodological backgrounds, including expertise in interprofessional collaboration and education (AW, female, medical student and doctoral student; MB, female, psychologist and medical doctoral student; MJW, male, nurse educator and PhD educational scientist; EB, female, nurse educator and PhD student; MSch, female, professor of health services research and psychologist), postgraduate medical education (MJW; EB; MS, male, professor of learning analytics), qualitative research (MJW, MSch), learning sciences (CR, female, PhD of learning sciences) and psychology (MS). These perspectives enabled contextual and analytical depth and supported critical reflection on attributions, hierarchies and routines across professional groups.

### 2.7 Trustworthiness

The quality of the research process was assessed against three criteria of qualitative research: transparency, intersubjectivity, and range (40, 41).

#### 2.7.1 Transparency

The case material was compiled from national and international guidelines. The elicitation guide was constructed according to Helfferich (32) and piloted. The analysis followed content-structuring qualitative content analysis (39) and was documented in a coding guide with category definitions and examples. All coding was carried out in MAXQDA, and the main coders (AW, MJW) kept memos throughout data collection and analysis. Supplementary Appendix A illustrates the analytic pathway from coded material to category summary, Supplementary Appendix C the framework used to assign DAs and IAs.

#### 2.7.2 Intersubjectivity

AW and MJW jointly coded two datasets to calibrate the application of the coding guide. The remaining material was coded by one researcher, and codes were iteratively discussed and refined in the research team using a consensus procedure (42). CAs, DAs, and IAs were assigned by consensus (MJW, CR). The category system was discussed in three rounds in a qualitative research workshop with researchers and practitioners from outside the project.

#### 2.7.3 Range

Each of the six experts analyzed three cases representing different patient histories and wound types, yielding 18 case-based datasets for within-case and cross-case comparison. Written responses and audio-recorded reflections provided two complementary forms of data. Data sufficiency was assumed when no substantially new aspects emerged. The findings rest on expert reflections on paper-based cases within the German health care system.

## 3 Results

### 3.1 Participants

The sample comprised two physicians working in inpatient care who are active in national and international wound care societies, two nurses working in higher education, and two pharmacists working in higher education and in clinical practice. All experts fully met the inclusion criteria stated above and are recognized experts in the field of wound care and research in Germany. Table 1 summarizes the characteristics of the participating experts.

**Table 1:** Characteristics of the participating experts (*n* = 6).

Expert	Profession	Practice setting	Professional experience (years)
A1	Physician	Inpatient care	10–15
A2	Physician	Inpatient care	10–15
P1	Nurse	Higher education (teaching)	10–15
P2	Nurse	Higher education (teaching)	16–20
PH1	Pharmacist	Higher education (professorship) and clinical practice	16–20
PH2	Pharmacist	Clinical practice	> 20

All experts were certified wound experts according to the standard of the Initiative Chronische Wunden (ICW), had teaching experience, had published scientific work in the field of wound research, and had prior experience with interprofessional collaborative practice and interprofessional education.

### 3.2 Overview of main and sub-categories

Table 2 shows the main category with relevant subcategories (SC) and coded text segments per subcategory.

**Table 2:** List of the main category with relevant subcategories.

Main category	Subcategory (SC)	Number of coded text segments
Critical points in collaboration	1. Pre-briefing outside the door	39
	2. Pain management	22
	3. Positioning	11
	4. Wound assessment and cleaning	30
	5. Selecting wound dressing and wound edge protection	21
	6. Wound documentation	23
	7. Patient education on wound care	11
	8. Debriefing and follow-up care	31

The eight critical points in collaboration reported here were formed inductively across all 18 datasets and were not prescribed by the material.

All main and sub-categories are listed below. Three subcategories are reported in full as examples, namely (SC 1) pre-briefing outside the door, (SC 4) wound assessment and cleaning, and (SC 8) debriefing and follow-up care. These three were selected because they contain the largest number of coded segments (39, 30 and 31, respectively), and because they represent the beginning, core and closing stages of the care process. This ensures that the framework's scope is covered. An allocation of CAs, DAs and IAs is shown. All subcategories, including the five not reported in full here, are documented in Supplementary Appendix B in table form with anchor quotations and marked at segment level with regard to the individual CAs, DAs and IAs.

### 3.3 Critical points in collaboration

#### 3.3.1 SC 1: “pre-briefing outside the door”

This code was assigned if the experts commented on what the students should discuss in advance to be able to carry out wound care.

The experts suggest an interprofessional preliminary discussion before each wound care session. All relevant information such as wound documentation, laboratory values, pain experiences from previous dressing changes as well as the clinical picture and its severity are discussed to create a common understanding (DAs: evidence generation and evaluation). Roles and responsibilities are clearly defined in the team, the planned procedure is jointly agreed, and alternatives are weighed up (IAs: intervention selection; intervention planning). During this discussion, particular attention is paid to the subsequent determination of measures such as dressing change intervals, the selection of suitable wound dressings and re-evaluation in the event of complications. The patient's experiences and needs are actively incorporated into the planning process.

“Here, the professions should exchange information on possible improvements with regard to dressing changes, intervals or the selection of wound dressings.” (pharmacist, PH1, pos. 9, CA: joint information processing)

The preliminary meeting on the doorstep thus serves as an interface between the knowledge and experience of the professions involved and a concrete action (CA: joint information processing). Structured planning strengthens the shared sense of responsibility and promotes the ability to act within the team, thereby enabling coordinated and effective care (CAs: regulation; coordination). Early coordination creates a shared understanding that increases patient safety and creates space for the integration of individual patient experiences and needs (CA: negotiating).

#### 3.3.2 SC 4: “wound assessment and cleaning”

This code was assigned if the experts commented on interprofessional interactions related to wound assessment (inspection of the old dressing and the wound) and wound cleaning.

Experts suggest ICP in wound assessment and cleaning. The inspection of the wound, the wound environment and the old wound dressing should be carried out as a team and systematically recorded and discussed (DA: evidence generation). The suitability of the old dressing for wound healing is critically assessed (IA: intervention evaluation). If there are any abnormalities or changes in the wound or dressing, the treatment concept is adjusted by the interprofessional team (IA: intervention evaluation and adjustment). This is followed by a discussion about the various wound cleaning methods, in which the advantages and disadvantages of each method are weighed up, considering the type and stage of the wound (IA: intervention generation and selection). The decision on wound cleaning and the selection of suitable products is also made in a joint discussion (IA: intervention selection).

“Discuss findings from the inspection: Color of exudate, amount, dressing exhausted, odor, contact with wound surface given?” (physician, A1, pos. 17, DA: evidence generation)

Wound assessment and cleaning are therefore interprofessional tasks in which clinical judgment, empirical knowledge and product-related expertise complement each other (CA: regulation). In a joint exchange, not only is the correct professional procedure coordinated, but a reflective process is initiated that makes uncertainties visible and promotes collective decision-making (CA: joint information processing). Precise wound care is thus achieved through the combination of specialist knowledge, shared responsibility and open communication (CA: negotiating).

#### 3.3.3 SC 8: “debriefing and follow-up-care”

This code was assigned if the experts commented on interprofessional interactions regarding the debriefing and further treatment of the wound.

Experts suggest an interprofessional debriefing of wound care to evaluate and optimize treatment (IA: intervention evaluation and adjustment). Reflecting on the effectiveness of the measures applied is of central importance. The next steps for further treatment should be jointly determined during the debriefing, as these require a comprehensive approach that includes both therapeutic measures (e.g., dressing change intervals) and the involvement of other professions (e.g., physiotherapy, pain therapy) (IA: intervention evaluation). Continuous monitoring is crucial to be able to react quickly to changes and adapt treatment accordingly (IA: intervention evaluation). Further treatment goes beyond regular wound care and also includes joint coordination of additional support such as smoking cessation, weight reduction or prostheses (IA: intervention generation). In addition, the patient's discharge to home, outpatient or long-term inpatient care must be coordinated (IA: intervention execution). A structured debriefing promotes consistency in the procedure and ensures that all professions involved are fully informed about the condition of the wound, the planned measures and long-term care.

“In the event of changes, consult the therapeutic team as quickly as possible. It may also require a therapy adjustment, which should be discussed in the interprofessional team and must take place as soon as possible. In the long term, a very, very close exchange is needed.” (nurse, P2, pos. 26, IA: intervention evaluation and adjustment)

Therefore, debriefing and further treatment are interprofessional tasks in which care is understood as an ongoing process with shared responsibility, not a completed action (CAs: joint information processing and regulation). Joint reflection on previous measures, necessary adjustments and further therapeutic steps forms the basis for interprofessional continuity of care, which is aimed at quality assurance, connectivity and patient-centered action (CAs: negotiation; coordination).

## 4 Discussion

This study examined wound care as an example of complex ICP to identify critical points in collaboration and analyze their functional structure. These accounts were analytically linked to the concept of noticing (11), the shared recognition of situations that require active interprofessional engagement. In the experts' accounts, noticing functioned as a trigger for collaborative activity, marking situations in which professionals needed to realign understanding, share knowledge, and coordinate decisions. Our analysis indicated that these moments can be systematically categorized into CAs (10, 11), DAs (9), and IAs (8). The resulting structure captures how reasoning and action are distributed and integrated across professional boundaries to manage clinical complexity. By specifying how such decisions are enacted through observable activities in interprofessional practice, these findings extend prior work, which has emphasized the context-dependent and socially embedded nature of management reasoning (21).

Across all phases of wound care, experts described a wide range of interprofessional reasoning and action. The theory-informed categorization of these activities into CAs, DAs, and IAs allowed us to reveal underlying functional structures that shaped how collaboration unfolded in context. The findings indicate that DAs and IAs are distinct yet closely intertwined and unfold in dynamic interplay with CAs, aligning with prior process-oriented findings (12) and extending them by showing how these interdependencies are structured through coordination processes in clinical practice. For example, information gathering, wound assessment, and evaluation of healing processes were identified as DAs, while selecting, implementing, and adapting treatment strategies were categorized as IAs. These were embedded in CAs such as joint information processing, negotiation, coordination, and regulation, which scaffolded the diagnostic and intervention-related decision processes. Consistent with prior work, these processes were not linear but iterative and continuously recalibrated through CAs in response to new information or changing patient needs (12). At the same time, the present findings clarify how these iterative dynamics are organized through context-specific coordination demands in clinical practice. Because activities were assigned at the level of synthesized category summaries rather than in temporal sequence, the present analysis was not intended to derive a fixed CA–DA–IA pathway. Future studies using sequentially coded interaction data could build on the present framework to examine whether recurring activity sequences emerge across collaboration points and clinical contexts.

The analysis highlights how familiar practices are functionally organized and interdependent. This may help explain why interprofessional collaboration often remains difficult in practice despite well-established guidelines: the challenge lies less in knowing what to do than in coordinating when and how different forms of reasoning and action need to be aligned.

These processes are not enacted uniformly. Experts differed in how strongly they emphasized early coordination, the timing of decision-making, and the degree to which responsibilities should be shared or profession-specific. These variations point to underlying tensions between standardization and flexibility, as well as between shared and profession-specific responsibility in interprofessional care.

By making these patterns explicit, the study contributes to practical approaches for improving collaboration in clinical care. The distinction between CAs, DAs, and IAs enables a more differentiated understanding of how interprofessional reasoning unfolds over time. Rather than treating collaboration as a general construct or background condition, the results highlight its structuring function in diagnostic and therapeutic decision-making.

### 4.1 Practical and educational implications of the CA–DA–IA lens

Against this background, the practical implications are twofold. First, the structure can help teams and institutions sharpen how existing collaborative routines are used in wound care. Practices like pre-briefings or debriefings are already established in many settings, but the CA–DA–IA lens can help focus them on the most critical content. For instance, pre-briefings can focus on diagnostic cues (DAs), planned interventions (IAs), and role clarification (CAs), while debriefing addresses intervention effectiveness (IAs), information gaps (DAs), and necessary team adjustments (CAs). This makes team coordination more targeted and transparent, especially in settings where time is limited and roles are distributed across professions.

Second, the structure provides a basis for developing and aligning training activities with real-world demands. Simulation-based scenarios can be designed around specific collaborative sequences, that require shared interpretation (CA) and coordinated decisions on diagnostic and intervention-related actions (DA, IA). Learners can also be asked to articulate how they reached joint decisions. This helps shift the focus from “working in parallel” to truly shared interprofessional reasoning. Immersive virtual reality provides one such environment. In a follow-up study, we developed a virtual reality simulation on the basis of the collaboration points reported here and tested it with medical, nursing and pharmacy students working on pressure ulcer management. Structured interprofessional virtual reality training improved procedural knowledge, whereas willingness to collaborate increased only after repeated exposure (23).

The CA–DA–IA lens can also guide feedback in clinical placements or simulation debriefings. Instead of general comments on teamwork, feedback can focus on whether DAs, IAs, and CAs were appropriately addressed. Making such activities visible helps learners understand what good interprofessional practice looks like and where concrete improvement is possible.

The findings point to two priorities for interprofessional curricula. First, they should name the points in a shared clinical task at which collaboration is decisive, instead of treating collaboration as a general competence that develops through joint attendance. Second, they should specify what each profession contributes at those points, because this would shift greater attention toward how DAs and IAs are allocated and coordinated under shared responsibility, than assuming that joint participation alone is sufficient to develop collaborative competence. The eight critical points identified here provide such a specification for wound care and can serve as a template for other shared clinical tasks.

Institutions can build on two elements of this study. The eight critical points can serve as learning objectives, specified by the descriptions and anchor quotations in Supplementary Appendix B. The CA–DA–IA structure provides the categories for observation and feedback instruments, and Supplementary Appendix C documents how diagnostic and intervention activities were assigned.

### 4.2 Assessment and future directions

Finally, the structure may support the development of assessment tools, especially in EPA-based formats. For instance, an EPA on “Perform wound monitoring and dressing care” could be enriched with observable markers such as “shares wound status and rationale for intervention,” “asks for input on product suitability,” or “negotiates adjustments in response to wound changes.” This allows educators to focus not only on clinical outcomes, but on the collaborative processes that shape them.

While this study focused on wound care, the identified patterns may also apply to other forms of complex interprofessional collaboration, such as discharge planning, tumor boards, or medication reconciliation (7, 11, 16). Future research may examine whether the CA–DA–IA structure applies across different contexts, which variants occur, and how noticing shapes collaborative dynamics in each case.

### 4.3 Limitations

The sampling strategy enabled access to highly qualified experts with extensive experience in clinical practice, teaching and research. However, prior exchange of views among participants cannot be fully excluded (36). Of the nine experts invited, six participated, a participation rate of 66.7%. The three experts who did not take part, one from each of the three professions, declined or did not respond because of time constraints and clinical workload, and all three target professions remained represented by two experts each. As no data were collected on the non-participants, a degree of self-selection cannot be excluded. The sample size (*n* = 6) reflects the qualitative design and does not allow generalization. Each expert completed three paper-based clinical patient cases, resulting in 18 analyses overall. Repetition across cases per expert supported within-case and cross-case reflections, contributing to dataset depth and robustness. Findings remain specific to the German health care context. However, the case material was compiled from national and international guidelines and the activity categories are drawn from established international frameworks, so the collaboration points are not tied to a single health system, even though the allocation of activities between professions may differ. Interpretive co-construction between experts and researchers shaped the analysis, consistent with principles of personalizing evaluation (37). Two of the 18 datasets were jointly coded to calibrate the coding framework, after which coding decisions were iteratively discussed within the research team and resolved through consensus. Because the analytic approach emphasized consensual interpretation rather than independent reliability estimation, no formal intercoder agreement coefficient was calculated. The synthesized findings were not returned to participants for member checking. However, interpretive credibility was supported through consensus discussions within the research team and three rounds of external discussion in a qualitative research workshop. The use of paper-based cases means that the study captures experts' anticipated reasoning about interprofessional collaboration rather than interaction as it unfolds in real time. The standardized, sequential case format nevertheless allowed experts from all three professions to reflect on the same clinical situations and enabled systematic comparison across cases and professional perspectives. The absence of time pressure, interruptions, and directly interacting team members may have elicited accounts closer to intended than enacted collaborative practice. Future observational or simulation-based studies can therefore build on the identified collaboration points and CA–DA–IA structure to examine how these activities unfold dynamically under authentic interactional constraints (for example, 12, 23).

## 5 Conclusion

This study provides a theory-informed and structured lens to analyze and support interprofessional collaboration in clinical care. The findings specify how DAs, IAs, and CAs are functionally related and how they structure interprofessional decision-making in context. In doing so, they complement existing process-oriented accounts of collaborative reasoning by providing a functional perspective on how these activities are organized in clinical practice.

This differentiation builds on existing clinical reasoning frameworks and extends them by foregrounding the collaborative mechanisms that enable coordinated action across professions. The resulting structure offers a practical foundation for the design of IPE formats. Future research should explore how this analytic approach can be applied across different care contexts and how it can best be integrated into teaching, supervision, and EPA-based evaluation.

## Data Availability

The original contributions presented in the study are included in the article/Supplementary Material, further inquiries can be directed to the corresponding author/s.

## 6 Previous presentations

Portions of this work were presented at the International Society of the Learning Sciences (ISLS) Helsinki, Finland.

## References

[1] Kaiser L Conrad S Neugebauer EAM Pietsch B Pieper D. Interprofessional collaboration and patient-reported outcomes in inpatient care: a systematic review. Syst Rev. (2022) 11(1):169. 10.1186/s13643-022-02027-x 35964148 PMC9375378

[2] Reeves S Pelone F Harrison R Goldman J Zwarenstein M. Interprofessional collaboration to improve professional practice and healthcare outcomes. Cochrane Database Syst Rev. (2017) 6(6):Cd000072. 10.1002/14651858.CD000072.pub3 28639262 PMC6481564

[3] Parker CN Johnston S Theobald KA. Promoting person-centered care for health baccalaureate students: piloting an interprofessional education approach to wound management. Adv Skin Wound Care. (2022) 35(10):1–8. 10.1097/01.ASW.0000873684.24346.be 36125457

[4] WHO World Health Organization. Framework for action on interprofessional education and collaborative practice. (2010). (Accessed 23.05.2024). Available online at: https://www.who.int/publications/i/item/framework-for-action-on-interprofessional-education-collaborative-practice

[5] Bartholomew N McKillop A Barr O. Approaching undergraduate interprofessional education (IPE) from a holistic program design perspective: an umbrella review. J Interprof Care. (2025) 40(2):400–16. 10.1080/13561820.2025.2582054 41368888

[6] Cadet T Cusimano J McKearney S Honaker J O’Neal C Taheri R. Describing the evidence linking interprofessional education interventions to improving the delivery of safe and effective patient care: a scoping review. J Interprof Care. (2024) 38(3):476–85. 10.1080/13561820.2023.2283119 38124506 PMC11009096

[7] Berger S Krug K Goetz K. Encountering uncertainty and complexity in decision-making: an observational study of clinical reasoning among medical and interprofessional groups of health care students. J Interprof Care. (2023) 37(2):262–71. 10.1080/13561820.2022.2061928 35687005

[8] Richters C Schmidmaier R Popov V Schredelseker J Fischer F Fischer MR. Intervention skills—a neglected field of research in medical education and beyond. GMS J Med Educ. (2024) 41(4):Doc48. 10.3205/zma001703 39415818 PMC11474644

[9] Fischer F Kollar I Ufer S Sodian B Hussmann H Pekrun R. Scientific reasoning and argumentation: advancing an interdisciplinary research agenda in education. FLR. (2014) 2(3):28–45. 10.14786/flr.v2i3.96

[10] Meier A Spada H Rummel N. A rating scheme for assessing the quality of computer-supported collaboration processes. IJCSCL. (2007) 2(1):63–86. 10.1007/s11412-006-9005-x

[11] Witti MJ Zottmann JM Wershofen B Thistlethwaite JE Fischer F Fischer MR. FINCA—a conceptual framework to improve interprofessional collaboration in health education and care. Front Med. (2023) 10:1213300. 10.3389/fmed.2023.1213300 PMC10577300 37849484

[12] Richters C Fischer M Fischer F Popov V. Team-based dynamics of diagnosis and intervention in a VR simulation: towards a reasoning process model. In: Oshima J Chen B Vogel F Järvelä S, editors. Proceedings of the 18th International Conference on Computer-Supported Collaborative Learning (CSCL 2025). Helsinki:International Society of the Learning Sciences (2025). p. 291–5.

[13] Boersema GC Smart H Giaquinto-Cilliers MGC Mulder M Weir GR Bruwer FA. Management of nonhealable and maintenance wounds: a systematic integrative review and referral pathway. Adv Skin Wound Care. (2021) 34(1):11–22. 10.1097/01.ASW.0000722740.93179.9f 33323798

[14] Brandt S Essig S Balthasar A. Health professionals’ willingness to share responsibility and strengthen interprofessional collaboration: a cross-sectional survey. BMC Med Educ. (2025) 25:102. 10.1186/s12909-024-06351-9 39838433 PMC11753034

[15] Spaulding EM Marvel FA Jacob E Rahman A Hansen BR Hanyok LA. Interprofessional education and collaboration among healthcare students and professionals: a systematic review and call for action. J Interprof Care. (2021) 35(4):612–21. 10.1080/13561820.2019.1697214 31865823 PMC7305974

[16] Richmond A Cooper N Gay S Atiomo W Patel R. The student is key: a realist review of educational interventions to develop analytical and non-analytical clinical reasoning ability. Med Educ. (2020) 54(8):709–19. 10.1111/medu.14137 32083744

[17] Fink MC Reitmeier V Stadler M Siebeck M Fischer F Fischer MR. Assessment of diagnostic competences with standardized patients versus virtual patients: experimental study in the context of history taking. J Med Internet Res. (2021) 23(3):e21196. 10.2196/21196 33661122 PMC7974754

[18] Kiesewetter J Fischer F Fischer MR. Collaborative clinical reasoning—a systematic review of empirical studies. JCEHP. (2017) 37(2):123–8. 10.1097/ceh.0000000000000158 28562501

[19] Radkowitsch A Fischer M Schmidmaier R Fischer F. Learning to diagnose collaboratively: validating a simulation for medical students. GMS J Med Educ. (2020) 37:1–12. 10.3205/zma001344 PMC7499460 32984510

[20] Cook DA Durning SJ Sherbino J Gruppen LD. Management reasoning: implications for health professions educators and a research agenda. Acad Med. (2019) 94(9):1310–6. 10.1097/ACM.0000000000002768 31460922

[21] Parsons AS Bryan K Morris C Durning SJ van Mook WNKA Ryan MS. Learning to manage: a qualitative exploration of how graduate medical trainees develop management reasoning. Acad Med. (2026) 101(4):411–21. 10.1093/acamed/wvaf068 41769763

[22] Mahler L Conrad P Busch D Erfurt-Berge C. Experts teaching wound management—a survey of current practices in Germany. Int Wound J. (2025) 22:e70694. 10.1111/iwj.70694 40419292 PMC12105872

[23] Bauer M Witti MJ Stadler M Weiß A Fischer MR Richters C. Interprofessional training in virtual reality for health care: experimental study on procedural knowledge and willingness to collaborate. JMIR Med Educ. (2026) 12(1):e85139. 10.2196/85139 42202475 PMC13215666

[24] Weiß A Richters C Kiriakidou R Hausen A Fischer MR Stadler M. The status of interprofessional medical and nursing education in wound care at German higher education institutions. GMS J Med Educ. (2026) 43(4):Doc47. 10.3205/zma001841 PMC13124531 42064281

[25] Ten Cate O Pool IA. The viability of interprofessional entrustable professional activities. Adv Health Sci Educ Theory Pract. (2020) 25(5):1255–62. 10.1007/s10459-019-09950-0 31872327

[26] O'Dowd E Lydon S O'Connor P Madden C Byrne D. A systematic review of 7 years of research on entrustable professional activities in graduate medical education, 2011–2018. Med Educ. (2019) 53(3):234–49. 10.1111/medu.13792 30609093

[27] Bozic U Witti MJ Schmidmaier R Fischer MR Zottmann JM Pudritz YM. Development of the entrustable professional activity “medication reconciliation” for clinical pharmacy. BMC Med Educ. (2024) 24(1):568. 10.1186/s12909-024-05504-0 38789955 PMC11127371

[28] Witti MJ Bozic U Hartmann D Kunisch R Pudritz Y Huber M. Definition of an entrustable professional activity for interprofessional patient handover for nurses—an interview study. Int J Health Prof. (2024) 11:75–85. 10.2478/ijhp-2024-0007

[29] Pool I Hofstra S van der Horst M Ten Cate O. Transdisciplinary entrustable professional activities. Med Teach. (2023) 45(9):1019–24. 10.1080/0142159X.2023.2170778 36708704

[30] Colson AR Cooke RM. Expert elicitation: using the classical model to validate experts’ judgments. REEP. (2018) 12(1):113–32. 10.1093/reep/rex022

[31] Tong A Sainsbury P Craig J. Consolidated criteria for reporting qualitative research (COREQ): a 32-item checklist for interviews and focus groups. Int J Qual Health Care. (2007) 19(6):349–57. 10.1093/intqhc/mzm042 17872937

[32] Helfferich C. The Quality of Qualitative Data Manual for Conducting Qualitative Interviews. Wiesbaden: Springer (2011).

[33] Leighton JP. Using Think-aloud interviews and Cognitive Labs in Educational Research. New York, NY, US: Oxford University Press (2017). p. 205.

[34] Soares M Colson A Bojke L Ghabri S Garay OU Felli JK. Recommendations on the use of structured expert elicitation protocols for healthcare decision making: a good practices report of an ISPOR task force. Value Health. (2024) 27(11):1469–78. 10.1016/j.jval.2024.07.027 39505473

[35] DiCicco-Bloom B Crabtree BF. The qualitative research interview. Med Educ. (2006) 40(4):314–21. 10.1111/j.1365-2929.2006.02418.x 16573666

[36] Przyborski A Wohlrab-Sahr M. Qualitative Social Research A Workbook. Munich:Oldenbourg Wissenschaftsverlag (2014).

[37] Patton M. How far dare an evaluator go toward saving the world?: redux, update, and a reflective practice facilitation tool. Am J Eval. (2021) 42:162–84. 10.1177/1098214020927095

[38] Saunders B Kitzinger J Kitzinger C. Anonymising interview data: challenges and compromise in practice. Qual Res J. (2015) 15(5):616–32. 10.1177/1468794114550439 PMC4582834 26457066

[39] Kuckartz U Rädiker S. Qualitative Content Analysis: Methods, Practice and Software. 2nd ed. London: SAGE Publications Ltd.; 2023. ISBN 978-1-5296-0913-4.

[40] Steinke I. Gütekriterien qualitativer forschung. In: Flick U von Kardorff E Steinke I, editors. Qualitative Forschung: Ein Handbuch. Reinbek bei Hamburg: Rowohlt Taschenbuch Verlag (2015). p. 319–31.

[41] Strübing J Hirschauer S Ayaß R Krähnke U Scheffer T. Gütekriterien qualitativer sozialforschung. Ein diskussionsanstoß. Z Soziol. (2018) 47(2):83–100. 10.1515/zfsoz-2018-1006

[42] Hill CE. Consensual Qualitative Research: A Practical Resource for Investigating Social Science Phenomena. Hill CE, editor. Washington, DC, US: American Psychological Association; 2012.

