# Supplementary material for: Making collaboration explicit through collaborative, diagnostic, and intervention activities: foundations for interprofessional education
Source: Front Med (Lausanne). 2026 Oct 23;13:1907531. doi: 10.3389/fmed.2026.1907531 (PMC13739574; doi:10.3389/fmed.2026.1907531)
Supplement: Supplementary file 1 [file Supplementaryfile1.pdf]

Appendix A *Example of analysis of the subcategory wound assessment and cleaning*

(1a) Transcript P1	(1b) Transcript PH2	(2) Explanation of the interpretation (memos)	(3) Conclusion	(4a) Summary Grid P1	(4b) Summary Grid PH2	(5) Summary table (P1-P2, A1-A2, PH1-PH2)
“This is once again very, very important and it is also important here to inspect not only the wound dressing, but also the wound as a whole, document this and report it.” (P1 Pos. 3)	*„During the ward round of the patient, the surrounding skin is assessed together and a joint decision is made about a suitable care product.”* (PH2 Pos. 7)	From the statements of P1 and PH2, it is concluded that it is important to inspect the wound, the surrounding area and the dressing and to discuss them together and document them.	Joint assessment, discussion, decision and documentation.	Importance of inspecting the dressing and the wound and discussing them together. Don`t forget the documentation.	Joint assessment of the wound and the surrounding skin and subsequent joint decision on suitable products.	Experts suggest ICP in wound assessment and cleaning. The inspection of the wound, the wound environment and the old wound dressing should be carried out as a team and systematically recorded and discussed. The suitability of the old dressing for wound healing is critically assessed. If there are any abnormalities or changes in the wound or dressing, the treatment concept is adjusted by the interprofessional team. This is followed by a discussion about the various wound cleaning methods, in which the advantages and disadvantages of each method are weighed up, considering the type and stage of the wound. The decision on wound cleaning and the selection of suitable products is also made in a joint discussion.

Note: The table shows: The transcripts (1a and 1b) are listed. This is followed by an excerpt from the memos (2) created, in which the researchers' thoughts and interpretations of the transcripts are recorded. In conclusion (3), the findings from the interpretations were summarised in summary grids (4a and 4b) for each interview. The column (5) summary table shows the summarized summary grids of all interviews.
