# Supplementary material for: Making collaboration explicit through collaborative, diagnostic, and intervention activities: foundations for interprofessional education
Source: Front Med (Lausanne). 2026 Oct 23;13:1907531. doi: 10.3389/fmed.2026.1907531 (PMC13739574; doi:10.3389/fmed.2026.1907531)
Supplement: Supplementary file 2 [file Supplementaryfile2.pdf]

Appendix B

Table of described subcategories

Subcategory (SC)	Code description	Anchor example	Interpretation
SC 1 Pre-briefing outside the door	This code was assigned if the experts commented on what the students should discuss in advance to be able to carry out wound care.<br><br>The experts suggest an interprofessional preliminary discussion before each wound care session. All relevant information such as wound documentation, laboratory values, pain experiences from previous dressing changes as well as the clinical picture and its severity are discussed to create a common understanding (DAs: evidence generation and evaluation). Roles and responsibilities are clearly defined in the team, the planned procedure is jointly agreed, and alternatives are weighed up (IAs: intervention selection; intervention planning). During this discussion, particular attention is paid to the subsequent determination of measures such as dressing change intervals, the selection of suitable wound dressings and re-evaluation in the event of complications. The patient's experiences and needs are actively incorporated into the planning process.	"*Here, the professions should exchange information on possible improvements with regard to dressing changes, intervals or the selection of wound dressings*" (PH1 pos. 9)	The preliminary meeting on the doorstep thus serves as an interface between the knowledge and experience of the professions involved and a concrete action (CA: joint information processing). Structured planning strengthens the shared sense of responsibility and promotes the ability to act within the team, thereby enabling coordinated and effective care (CAs: regulation; coordination). Early coordination creates a shared understanding that increases patient safety and creates space for the integration of individual patient experiences and needs (CA: negotiating).

SC 2 Pain management	This code was assigned if the experts commented on interprofessional interactions related to pain management.<br>The experts suggest interprofessional collaboration in pain management. This includes a structured pain assessment (DA: evidence generation), which involves collecting pain history and regular re-evaluation in order to adjust care as required (IA: intervention evaluation). Pain therapy is discussed and carried out jointly as a team: the physician prescribes the medication, the pharmacy checks this and suggests alternatives if there are any restrictions (IA: intervention selection). Nursing staff administer the medication and monitor the progress (time of last pain medication administration before dressing change) (IA: intervention execution and intervention evaluation). Cooperation between professions ensures the continuous optimisation of pain therapy during wound care and between dressing changes (IA: intervention adjustment). This structured exchange significantly contributes to effective pain management and successful wound care despite pain.	*“Pain management is a collaborative effort: prescription, adjustment by physician, medication administration* + *monitoring of progress by nursing staff (+ physician), consultation with pharmacy, Medicheck pharmacy“* (P1 pos. 13)	Pain management is therefore an interprofessional task in which medical, nursing, and pharmaceutical expertise work together in a targeted manner (CA: joint information processing). Continuous exchange within the team strengthens shared responsibility for effective, patient-centred pain therapy, enabling dynamic adaptation to individual needs and clinical changes (CA: regulation). This close coordination ensures high-quality wound care, even in painful conditions.
SC 3 Positioning	This code was assigned if the experts commented on interprofessional interactions related to positioning.<br>The experts suggest interprofessional collaboration when positioning the patient to ensure optimal positioning for wound care. Possible positioning options are discussed together (IA: intervention generation) and	*“Correct positioning in relation to the patient and the bed at working height is a very important aspect. It is particularly important to work together in interdisciplinary or joint general teams.”* (A2 pos. 22)	The positioning of patients is therefore a coordinated, interprofessional process that aims to provide patient-centered care and physically less demanding work for the team (CA: Coordination). By consciously coordinating positioning, role allocation, and working height, conditions are

	agreed upon as a team (IA: intervention selection) to avoid pressure points and pain for the patient. Careful coordination is required to adjust the bed to working height and position staff around the patient (IA: intervention execution). In addition, positioning should be reevaluated during and after wound care (IA: intervention evaluation) and adjusted if necessary (IA: intervention adjustment). For complex dressing changes or necessary positioning changes, at least two people should work together as a team.<br>.		created that take into account both the well-being of patients and the needs of the team (CA: Coordination).
SC 4 Wound assessment and cleaning	This code was assigned if the experts commented on interprofessional interactions related to wound assessment (inspection of the old dressing and the wound) and wound cleaning.<br><br>Experts suggest ICP in wound assessment and cleaning. The inspection of the wound, the wound environment and the old wound dressing should be carried out as a team and systematically recorded and discussed (DA: evidence generation). The suitability of the old dressing for wound healing is critically assessed (IA: intervention evaluation). If there are any abnormalities or changes in the wound or dressing, the treatment concept is adjusted by the interprofessional team (IA: intervention evaluation and adjustment). This is followed by a discussion about the various wound cleaning methods, in which the advantages and	„*Discuss findings from the inspection: Color of exudate, amount, dressing exhausted, odor, contact with wound surface given?"* (A1 pos. 17)	Wound assessment and cleaning are therefore interprofessional tasks in which clinical judgment, empirical knowledge and product-related expertise complement each other (CA: regulation). In a joint exchange, not only is the correct professional procedure coordinated, but a reflective process is initiated that makes uncertainties visible and promotes collective decision-making (CA: joint information processing). Precise wound care is thus achieved through the combination of specialist knowledge, shared responsibility and open communication (CA: negotiating).

	disadvantages of each method are weighed up, considering the type and stage of the wound (IA: intervention generation and selection). The decision on wound cleaning and the selection of suitable products is also made in a joint discussion (IA: intervention selection).		
SC 5 Selecting wound dressing and wound edge protection	This code was assigned if the experts commented on interprofessional interactions related to the selection of wound dressings and wound edge protection.<br><br>Experts suggest an interprofessional discussion on the selection of wound dressings and wound edge protection in order to make an informed and targeted decision that is tailored to the individual needs of the patient (IA: Intervention selection). All relevant characteristics of the wound, such as wound type, size, exudate level, and stage of wound healing, should be taken into account (DA: Evidence Evaluation). It is equally important to weigh the effectiveness, workload, and cost-effectiveness of the products used (IA: Intervention selection) in order to ensure optimal and economical wound care.	*„Reference to economic aspects when selecting wound dressings, decision based on wound type/size, exudation, stage of wound healing, economic aspects“* (PH1 pos. 32)	The selection of wound dressings and wound edge protection is therefore an interprofessional task in which clinical effectiveness, practical feasibility, and economic responsibility must be weighed up (CA: regulation). This decision-making process clearly shows that professional action goes beyond purely medical indications and is characterized by prioritization and a variety of different perspectives. The decision is thus an expression of the joint effort to combine quality, efficiency, and individuality in wound care (CA: negotiating).
SC 6 Wound documentation	This code was assigned if the experts commented on interprofessional interactions related to the wound documentation.	*„This is about documentation. It is important to have a standardized procedure within the team based*	Wound documentation is therefore an interprofessional task that requires a high degree of accuracy, standardization, and coordination to ensure complete and precise recording of wound

	Experts suggest that wound documentation should be carried out by an interprofessional team to ensure that wound care is recorded completely and accurately. Part of the documentation (wound condition) should be completed when the wound status is assessed (DA: Evidence generation) to avoid having to add information later from memory. Further documentation is then carried out after treatment (IA: Intervention Execution). A standardized procedure based on wound documentation forms is recommended, which is coordinated with the practical procedure (IA: Intervention planning). The documentation includes both written and photographic records (IA: Intervention execution). Photographic documentation, especially for areas of the body that are difficult to access, such as the heel, presents a particular challenge. Interprofessional discussion and implementation of this photographic documentation is therefore essential to ensure that the images meet the required standards and that the wound is correctly recorded (DA: Evidence evaluation). All team members must be proficient in wound documentation to ensure complete and traceable documentation of wound care (IA: Intervention planning).	*on defined wound documentation forms."* (P1 pos. 3)	care (CAs: coordination; joint information processing). It goes beyond the mere recording of information and becomes an integral part of care. This ensures the quality and traceability of therapeutic actions (CA: regulation). Shared responsibility for written and photographic documentation strengthens transparency within the team and commitment to the care goal (CA: coordination).
SC 7 Patient education on wound care	This code was assigned if the experts commented on interprofessional interactions related to wound care education for patients.	*„Particularly important here: Explain to patients what the risks are, how an infection manifests itself, what to do in such a case, and*	Informing patients and their relatives about the correct way to treat the wound is therefore an interprofessional task in which different professional perspectives are brought together in a

	Experts suggest interprofessional collaboration in providing patients with information on wound care to ensure comprehensive and consistent education (IA: Intervention planning). All professional groups should work together to educate the patient, as each profession contributes specific knowledge and different perspectives (IA: Intervention execution). It is particularly important to educate patients about the dangers of infections, their symptoms, and what to do in the event of an infection (IA: Intervention execution). Patients and their relatives should be actively involved in wound care in order to learn the necessary steps (IA: Intervention execution). A consistent approach and written information materials are essential in order to provide patients with clear and understandable information about treatment, preventive measures, and safe wound care at home (IA: Intervention planning and intervention execution).	*where to turn for help"* (A1 pos. 11)	joint support process (CA: joint information processing). It is important to share responsibility for ensuring that knowledge is communicated in a comprehensible, consistent, and structured manner (CA: coordination). This makes the involvement of patients in their care a central area of interprofessional cooperation, promoting participation, self-efficacy, and continuity of care.
SC 8 Debriefing and follow-up care	This code was assigned if the experts commented on interprofessional interactions regarding the debriefing and further treatment of the wound.<br><br>Experts suggest an interprofessional debriefing of wound care to evaluate and optimize treatment (IA: intervention evaluation and adjustment). Reflecting on the effectiveness of the measures applied is of central importance. The next steps for further treatment	*„In the event of changes, consult the therapeutic team as quickly as possible. It may also require a therapy adjustment, which should be discussed in the interprofessional team and must take place as soon as possible. In the long term, a very, very*	Therefore, debriefing and further treatment are interprofessional tasks in which care is understood as an ongoing process with shared responsibility, not a completed action (CAs: joint information processing and regulation). Joint reflection on previous measures, necessary adjustments and further therapeutic steps forms the basis for interprofessional continuity of care, which is aimed

	should be jointly determined during the debriefing, as these require a comprehensive approach that includes both therapeutic measures (e.g. dressing change intervals) and the involvement of other professions (e.g. physiotherapy, pain therapy) (IA: intervention evaluation). Continuous monitoring is crucial to be able to react quickly to changes and adapt treatment accordingly (IA: intervention evaluation). Further treatment goes beyond regular wound care and also includes joint coordination of additional support such as smoking cessation, weight reduction or prostheses (IA: intervention generation). In addition, the patient's discharge to home, outpatient or long-term inpatient care must be coordinated (IA: intervention execution). A structured debriefing promotes consistency in the procedure and ensures that all professions involved are fully informed about the condition of the wound, the planned measures and long-term care.	*close exchange is needed." (P2 pos. 26)*	at quality assurance, connectivity and patient-centered action (CAs: negotiating; coordination).

Note: The table shows: The subcategories (1) identified during the analysis. This is followed by the code descriptions (2). An exemplary anchor quote (3) from the expert interviews provides context. The fourth column (4) presents the interpretation of the respective subcategory.
