# Supplementary material for: Making collaboration explicit through collaborative, diagnostic, and intervention activities: foundations for interprofessional education
Source: Front Med (Lausanne). 2026 Oct 23;13:1907531. doi: 10.3389/fmed.2026.1907531 (PMC13739574; doi:10.3389/fmed.2026.1907531)
Supplement: Supplementary file 3 [file Supplementaryfile3.pdf]

Appendix C

Analytic framework used to categorize DAs and IAs in the summarized expert material

	Cognitive Activity	Definition	Assignment in this Study (Summary Segments)	Behavioral Markers for Future Transcription
DAs	Evidence Generation	Contributing or retrieving clinically relevant information to understand the patient's condition.	Documentation, reporting, or recording of patient-related data without interpretation was described.	Clinician describes symptoms, test results, wound status, or patient experiences without making judgments (e.g., "wound has yellow discharge").
	Evidence Evaluation	Interpreting or critically judging the diagnostic relevance of information to better understand the patient's condition or problem.	Interpretation or discussion of the meaning, severity, or implications of clinical information was mentioned.	Clinician interprets findings (e.g., "that discharge could indicate infection"; "pain level is concerning").
IAs	Intervention Generation	Developing a range of reasonable treatment strategies or options to address a clinical problem or goal, even in cases of diagnostic uncertainty.	Treatment options or supportive measures were brought up without referencing selecting or comparing of treatment options.	Clinician introduces possible options (e.g., "we could use foam dressing"; "compression might be useful").
	Intervention Selection	Weighing, contrasting, prioritizing, and selecting interventions based on feasibility, effectiveness, context, and individual patient needs.	Summary segments involved weighing or prioritizing options, or when a choice between them was described.	Clinician compares or selects interventions (e.g., "foam is better here"; "let's use saline instead").
	Intervention Planning	Structuring the implementation of an intervention by defining treatment goals, setting monitoring parameters and thresholds, sequencing steps, and assigning	Summary segments involved defining the sequence, monitoring, or responsibilities of a chosen intervention.	Clinician defines monitoring plan or steps (e.g., "change dressing every 2 days"; "check progress weekly").

	responsibilities while anticipating patient response over time.		
Intervention Executing	Carrying out or initiating the planned intervention, including organizing, performing, and coordinating clinical procedures or actions.	Summary segments indicated that an intervention or care action is being carried out.	Clinician performs action (e.g., applies dressing, administers medication, arranges discharge).
Intervention Evaluation	Assessing the effectiveness or appropriateness of an intervention based on the patient's response or clinical progression.	Summary segments referred to reflection on whether treatment was effective or required revision.	Clinician reflects on effects (e.g., "the wound improved with this dressing"; "pain has decreased").
Intervention Adjustment	Modifying an intervention or its implementation based on evaluation outcomes or deviations from expected clinical progress.	The need to adjust treatment based on observed changes was mentioned.	Clinician adjusts intervention (e.g., "we'll switch dressings"; "increase frequency of dressing changes").

Note. While all activity assignments in this study were made at the level of synthesized category summaries, the column "Behavioral Markers for Future Transcription" provides indicative behavioral markers that may guide future research aiming to code these activities directly in behavioral data (e.g., transcribed simulation sessions or real-world clinical team interactions).
